# Environmental microbiome in the home and daycare settings during the COVID‐19 pandemic, and potential risk of non‐communicable disease in children

**DOI:** 10.1111/1758-2229.13233

**Published:** 2024-01-12

**Authors:** Jill A. McKay, Matthew Crown, Matthew Bashton, David Pearce, Jane A. Entwistle, Vartul Sangal

**Affiliations:** ^1^ Faculty of Health and Life Sciences Northumbria University Newcastle upon Tyne UK; ^2^ Faculty of Environment and Engineering Northumbria University Newcastle upon Tyne UK

## Abstract

An exposure to diverse microbial population early in life is important for the development of immunity against various non‐communicable diseases including asthma, childhood leukaemia and other cancers. Social mixing in daycare settings helps with exposure to a variety of microbes. However, social isolation and a high emphasis on workplace hygiene during the COVID pandemic may have affected children's exposure to diverse microbiota. The structure of microbial communities and their role in developing immunity to various diseases are not well understood. In this study, we investigated the structure of microbial communities in daycare and home settings during the pandemic. Interestingly, microbial diversity was relatively higher in dust samples collected from homes, with human‐associated taxa being more prevalent compared to those from daycare settings. Environmental microbes were more abundant in dust samples from daycare providers. These results potentially suggest that cleaning practices during the pandemic may have influenced the diversity and microbial abundance of the daycare samples. Several bacterial taxa detected in both the environments are known to induce anti‐inflammatory and immunomodulatory responses, conferring protection from various diseases. Therefore, exposure to diverse microbial population in early childhood may play an important role in developing immunity against various non‐communicable and infectious diseases.

## Graphical abstract

Home dust samples contained higher microbial diversity with human‐associated taxa being prevalent than daycare samples where environmental taxa were more common. Several taxa detected in both the environments are known to induce immune responses, potentially conferring protection from various non‐communicable and infectious diseases among children.

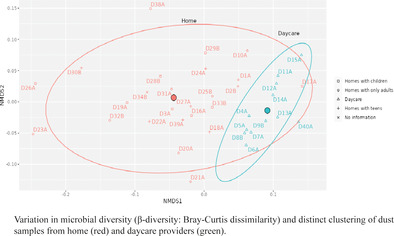

## INTRODUCTION

Limited exposure to microbes in early life due to increased levels of hygiene and a low microbial burden within the environment is a limiting factor in the development of the immune system which increases the risk of some non‐communicable diseases (NCDs; Bach, 2002; Bach & Chatenoud, 2012; Greaves, 2018; Greaves & Buffler, 2009). In particular, epidemiological evidence suggests the development of asthma, childhood leukaemia and other childhood cancers may be aetiologically linked to a lack of microbial exposure in early life (Ajrouche et al., 2015; Lupo et al., 2014; Marcotte et al., 2014; Peppas et al., 2023; Pombo‐de‐Oliveira et al., 2023; Rudant et al., 2015; Urayama et al., 2011). The composition of the infant microbiome changes rapidly in the early years (Isolauri, 2012) and can be influenced by a range of factors (Madan et al., 2012; Munyaka et al., 2014), including environmental cues (Edmonds‐Wilson et al., 2015; Xu & Knight, 2015). Environmentally induced differences in the infant microbiome, especially reduced diversity of certain taxa, may lead to altered and persistent microbial profiles. This in turn may influence normal physiology and therefore, the risk of developing childhood diseases, including asthma and leukaemia (Chiu et al., 2017; Hilty et al., 2010; Rajagopala et al., 2016; Wang et al., 2014), but may also extend into adulthood influencing the risk of other NCDs, including irritable bowel disease and Crohn's disease (Manichanh et al., 2006; Ott et al., 2004; Scher et al., 2015).

Social mixing is often used as a surrogate measure of microbial exposure which captures asymptomatic exposure and minor infections likely to go unreported or recalled (Greaves & Buffler, 2009). Daycare attendance offers social mixing at an important stage of immune system development and is widely accepted as a strong predictor for increased microbial exposure and infection during infancy and childhood (Holmes et al., 1996; Osterholm, 1994). The combination of immature immune systems and lack of appropriate hygiene behaviours in infants is believed to promote not only infection but also the transmission of bacterial commensals (Urayama et al., 2008). Furthermore, daycare attendance as a proxy measure of microbial exposure has been inversely associated with the incidence of childhood leukaemia (Ajrouche et al., 2015; Rudant et al., 2015; Urayama et al., 2011). Exposure to microorganisms in the outdoor environment has been found to increase microbial diversity on skin and in the nasal cavity (Selway et al., 2020). However, there have been limited attempts to compare microbial diversity and composition across daycare and home environments to identify microbes that may be key in conferring such protection. In this study, we have analysed microbial diversity in dust samples collected from homes and daycare providers to identify microbes that are more prevalent in the daycare settings and may reduce the risk of childhood diseases.

## MATERIALS AND METHODS

### Sample collection

Vacuum bags or subsamples were collected as previously explained (Thompson et al., 2021). In total, 12 dust samples were collected from three local daycare providers, two provided five sequential samples a month apart between March and July 2021 and the third daycare provided two samples in June and July 2021. Twenty‐five dust samples were collected from homes, mostly from colleagues working at Northumbria University between April and July 2022. Similar to the previous study, home sample providers (participants) completed a short survey providing some information on the occupants, including number, gender and ages of occupants (Thompson et al., 2021).

### DNA extraction and amplicon sequencing

The samples were sieved through a UV‐sterilized 250 μm single‐use nylon mesh to remove larger particulates and fibrous material in a Class II biosafety cabinet. Microbial DNA was extracted from 1 g dust using DNeasy PowerSoil Pro Kit (Qiagen). 16S rRNA amplicon sequencing was carried out using the MiSeq platform as previously described (Thompson et al., 2021).

### Data analyses

16S amplicon sequencing reads were pre‐processed by BBDuk and TrimGalore (Krueger et al., 2021) and read pairs <240 bp after trimming were removed. Taxonomy assignment was carried out using Kraken2 (Lu & Salzberg, 2020) and Bracken (Lu et al., 2017) packages against SILVA v138.1 database (Quast et al., 2013; Yilmaz et al., 2014). A custom Bracken database with sequences of optimum read length of 250 bp was constructed for re‐estimation of abundance at the genus level. A minimum of 10 reads were required for retaining taxa assignments at the genus level. If this threshold was not met, reads were redistributed probabilistically to other genera.

Operational taxonomic unit (OTU) tables were analysed using various R packages including decontam (Davis et al., 2018), ggplot2 (Villanueva & Chen, 2019), microbiome (Lahti & Shetty, 2017), phyloseq (McMurdie & Holmes, 2013), tidyverse (Wickham et al., 2019) and vegan (Oksanen et al., 2020). Computational decontamination was performed by decontam package (Davis et al., 2018) following the frequency based statistical identification method with concentration and extraction (negative) control data. There was relatively low support for any contaminating taxa to be excluded, so no taxa were removed from downstream analysis.

The rarefaction method was used for determining the minimum sample depth for further analysis. All samples in the rarefaction curve have plateaued at 40,952 reads (Figure S1), suggesting that sequences have captured most of the diversity present in samples, and the rarefaction curve has reached saturation. On this basis, all samples can be included in the downstream analysis. To establish the precision of sample sequencing, technical replicates were included for sequencing and were investigated using the Bray–Curtis dissimilarity NMDS ordination. Technical replicates clustered closely together (Figure S2) confirming the reproducibility of the dataset and therefore, only a single replicate with the highest sequencing depth was used for downstream statistical analyses.

Shannon and inverse Simpson alpha diversities were calculated using the vegan R package (Oksanen et al., 2020) and normalcy of data was examined using a Shapiro–Wilk test. Beta diversity analysis was performed using the avgdist function from the vegan R package. Diversity was computed 100 times using subsamples at 40,952 reads and the final diversity for each sample used in analysis was the mean of these iterations. Diversities were visualized with two‐dimension NMDS ordination with centroids calculated as mean diversities and visualized using stat_ellipse from within the ggplot2 package. ADONIS2 (Permutational Multivariate Analysis of Variance using Distance Matrices) was used to test differences in beta‐diversities between the groups and betadisper to test for differences in dispersion using analysis of variance (ANOVA). Core taxa investigation was performed using a taxon prevalence threshold of 75% at a 1% detection rate. This threshold has been previously used to determine core taxa (Thompson et al., 2021). To explore the effect of location type on beta diversity, SIMPER analysis was used to identify the contribution of individual taxa to genus level Bray–Curtis beta diversity using pairwise comparison of the home versus daycare data set and statistically significant differences in abundance between homes and daycare were determined by Kruskal–Wallis rank sum testing with FDR‐adjustment. Additionally, LEfSe analysis (Segata et al., 2011) was performed with the default settings using Biobakery (McIver et al., 2018) at the Galaxy server (The Galaxy Community, 2022).

## RESULTS AND DISCUSSION

### Microbial abundance and diversity among samples from homes and daycare providers

**FIGURE 1:**
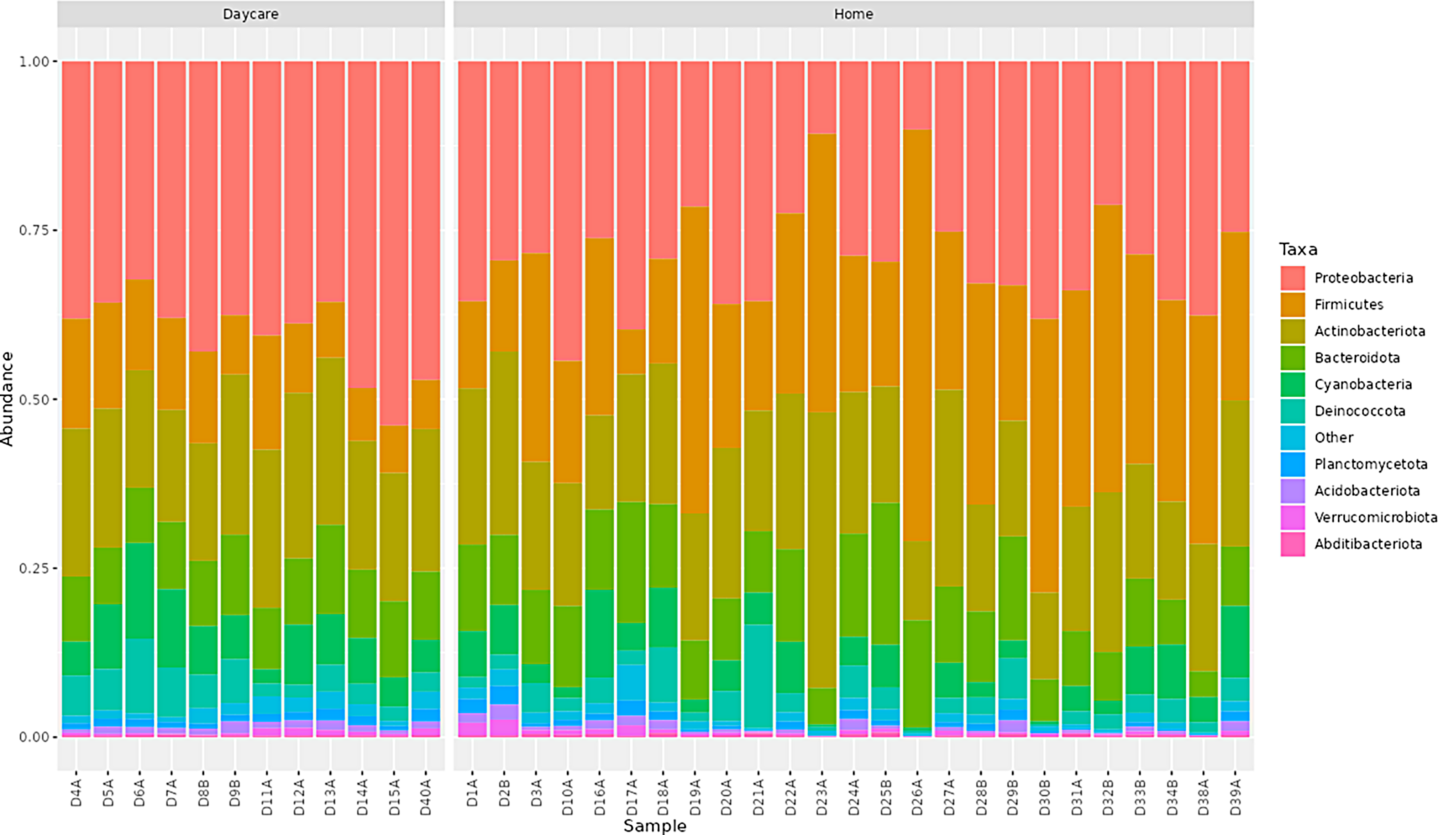
Composition of the dust samples from homes and daycares at the phylum level. Ten most prevalent phyla are displayed, and the remaining phyla are grouped as ‘other’.

**FIGURE 2:**
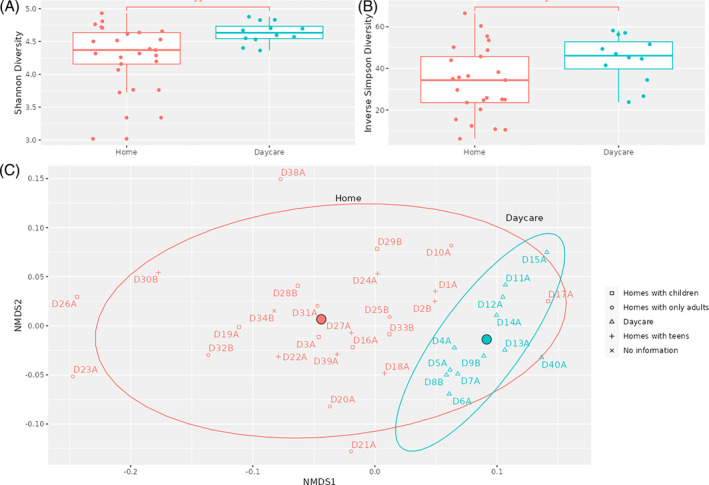
Alpha diversity among samples from home and daycare settings using (A) Shannon and (B) Inverse Simpson diversity indices; (C) NMDS ordination of sample β‐diversity (Bray–Curtis dissimilarity). Samples are coloured by location type, with means highlighted and centroids drawn indicating 95% CI. CI, confidence interval.

Microbial diversity and composition of taxa varied between home and daycare samples. *Actinobacteriota*, *Bacteriodota*, *Firmicutes* and *Proteobacteria* were the most prevalent phyla accounting for >70% abundance among the samples, both from the home and daycare environments (Figure 1). Other common phyla observed in these samples included *Abditibacteriota*, *Acidobacteriota*, *Cyanobacteria*, *Deinococcota*, *Planctomycetota* and *Verrucomicrobiota* (Figure 1). However, microbial diversity among the home samples was significantly higher than the daycare samples (Shannon *p* = 0.007, inverse Simpson *p* = 0.0117). The samples from daycare clustered away from the home samples (Figure 2; ADONIS2, *p* value = 0.001). ADONIS2 analysis may be sensitive to dispersion effects; therefore, differences in dispersion were also tested using ANOVA (betadisper, *p* value = 0.006). These results suggest that microbial communities in the dust samples from homes are more diverse and quite distinct from those of the samples from the daycare providers. In previous studies, microbial diversity in the daycare settings was found to be higher in comparison to the outdoor environment (Beasley et al., 2022), which was influenced by the level of occupancy and the climate (Estensmo et al., 2021). Remarkably, higher diversity was observed among the home samples despite the numbers of occupants being much lower than in the daycare settings in this study. These samples were collected during the COVID pandemic, and we believe that more robust hygiene strategy and frequent cleaning in the daycare settings during the pandemic is likely to be responsible for the lower diversity observed in this study.

Eight dust samples were collected from homes with children aged 0–10 years, eight with children/teenagers aged 11–18 years (classed as teen) and nine with all adult occupants (Table S1). However, we did not see any sub‐clustering of the home samples based on the age of occupants. This is consistent with the previous observation that the built environments inhabited by adults and young children are functionally equivalent (Beasley et al., 2022). Our study found significant variations in the microbial diversity and composition of taxa between home samples and the daycare providers, potentially reflecting the differences in children's interaction with the environment in the daycare settings such as outdoor activities.

### Core and variable microbiome between homes and daycare settings

Several genera, including *Acinetobacter*, *Bacillus*, *Corynebacterium*, *Flavobacterium*, *Hymenobacter*, *Lactobacillus*, *Nakamurella*, *Pseudomonas* and *Streptococcus*, were detected among all samples from home and daycare environments (Figure S3). The species belonging to some of these taxa have been isolated from diverse hosts and environments. For example, various species within genus *Corynebacterium* have been isolated from animals, humans, marine and terrestrial environments including coral, river water, marine sediment, sand, soil and sewage (Dover et al., 2021). This genus also includes significant pathogens such as *Corynebacterium diphtheriae*, *Corynebacterium pseudotuberculosis* and *Corynebacterium ulcerans* that can cause life‐threatening diphtheria or diphtheria‐like infections in humans and animals (Sangal & Burkovski, 2020). Several members of the genera, such as *Acinetobacter, Corynebacterium*, *Flavobacterium*, *Staphylococcus* and *Streptococcus*, are common human skin microflora (Beasley et al., 2022; Byrd et al., 2018), but some of them are opportunistic pathogens with abilities to cause severe infections (Flowers & Grice, 2020).

The taxa *Acinetobacter*, *Hymenobacter*, *Nocardioides*, *Pseudomonas*, *Rubellimicrobium*, *Sphingomonas*, *Spirosoma* and *Truepera* were common between the home and daycare samples that were present in at least 75% samples with ≥1% abundance. These taxa were also reported to be core in the United Kingdom home dust samples previously (Thompson et al., 2021) except for *Pseudomonas*. *Bacillus*, *Corynebacterium*, *Staphylococcus* and *Streptococcus* were the unique core taxa in the home samples, whereas *Aliterella*, an uncultured *Chroococcidiopsaceae*, *Deinococcus* and *Nakamurella* were unique to the daycare samples. Notably, cyanobacteria including *Aliterella* and uncultured *Chroococcidiopsaceae* were common to all daycare samples. *Aliterella* strains have been isolated from cyanolichens and stones of the arid Atacama Desert (Jung et al., 2020; Jung et al., 2021), whereas *Chroococcidiopsaceae* species have been found in clouds, rainwater and urban greenspaces (Dillon et al., 2020; Mhuireach et al., 2021). *Deinococcus* species are often isolated from extremophilic environments and are well known for resistance to diverse environmental stress conditions (Gerber et al., 2015). *Deinococcus* has also been identified in daycare facilities, previously (Andersson et al., 1999). It is possible that outdoor activities organized by the daycare providers have contributed to the presence of these taxa in the dust samples.

**FIGURE 3:**
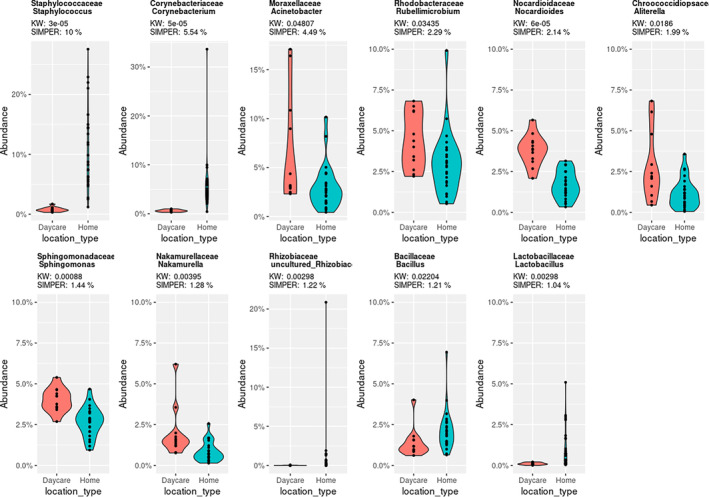
Violin plots showing the taxa with significant differences in their abundances between the home and daycare samples. SIMPER and Kruskal–Wallis (KW) values are displayed for each taxon.

**FIGURE 4:**
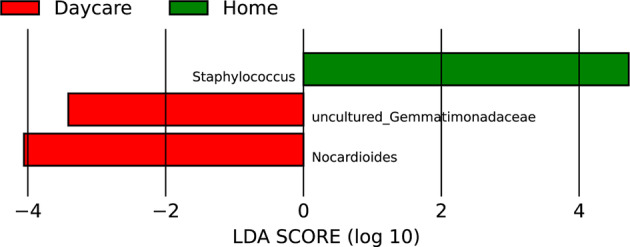
Most prevalent taxa differentiating the dust samples from daycare and home.

Twenty‐one taxa were noted for >1% variation in their abundance between the home and the daycare dust samples (Table 1; Figure 3). The difference in the abundance of *Flavobacterium*, *Lactococcus*, *Paracoccus*, *Prevotella*, *Pseudomonas*, *Psychrobacter*, *Streptococcus*, *Truepera* and uncultured *Chroococcidiopsaceae* was not significant and the difference in the abundance of *Acinetobacter*, *Aliterella*, *Bacillus* and *Rubellimicrobium* was only marginally significant (*p* values between 0.019 and 0.048) between the two environments (Table 1). *Corynebacterium*, *Staphylococcus*, *Lactobacillus* and uncultured *Rhizobiaceae* were significantly more abundant in the home dust than in daycare samples, whereas *Nakamurella*, *Nocardioides*, *Sphingomonas* and *Spirosoma* were more abundant in the latter (Table 1, Figure 3). These findings are also supported by the LEfSe analysis, where environment‐associated taxa (*Nocardioides* and uncultured *Gemmatimonadceae*) differentiated daycare samples from home samples (Figure 4). Human‐associated taxon *Staphylococcus* was found to be prevalent among the home samples (Figure 4). The higher abundance of human‐associated genera, including *Corynebacterium*, *Staphylococcus* and *Lactobacillus* (Byrd et al., 2018; Thompson et al., 2021) among the home dust samples was surprising as these were expected to be equally prevalent in the daycare settings (Beasley et al., 2022). As mentioned above, daycare samples were collected during the COVID pandemic with very high emphasis on personal hygiene including frequent hand washing and use of sanitizers, and more robust cleaning practices of the shared spaces, which were unlikely to have been as prevalent in the home settings. Also, most of the daycare samples were collected in 2021 under stricter pandemic hygiene guidelines, whereas the home samples were collected in 2022 when guidelines were more relaxed. Most of the daycare samples were collected during the spring or summer time when weather for outdoor activities is relatively more favourable and may have resulted in higher prevalence of environment‐associated taxa such as *Nakamurella* and *Nocardioides* (Nouioui et al., 2017; Prauser, 1976).

**TABLE 1:** SIMPER analysis of taxa at genus level that are responsible for >1% variation between home and daycare samples.

SIMPER	OTU	Fdr krusk pval	Home abundance	Home SD	Daycare abundance	Daycare SD
0.100	*Staphylococcus*	0.00003	0.109	0.070	0.008	0.004
0.055	*Corynebacterium*	0.00005	0.062	0.062	0.006	0.002
0.044	*Acinetobacter*	0.04807	0.030	0.023	0.064	0.055
0.024	*Truepera*	0.20201	0.020	0.026	0.031	0.025
0.023	*Lactococcus*	0.06117	0.028	0.038	0.010	0.010
0.023	*Rubellimicrobium*	0.03435	0.030	0.020	0.044	0.017
0.021	*Nocardioides*	0.00006	0.017	0.008	0.038	0.010
0.020	*Aliterella*	0.01860	0.012	0.009	0.028	0.021
0.019	Uncultured *Chroococcidiopsaceae*	0.92246	0.025	0.019	0.022	0.013
0.017	*Psychrobacter*	0.74405	0.014	0.028	0.010	0.015
0.017	*Streptococcus*	0.92246	0.023	0.017	0.022	0.015
0.016	*Pseudomonas*	0.69080	0.022	0.017	0.023	0.012
0.015	*Paracoccus*	0.69080	0.009	0.010	0.014	0.025
0.015	*Sphingomonas*	0.00088	0.027	0.009	0.040	0.007
0.014	*Spirosoma*	0.74405	0.023	0.014	0.025	0.008
0.013	*Bacillus*	0.02204	0.021	0.013	0.014	0.009
0.012	*Prevotella* 9	0.08980	0.014	0.025	0.001	0.001
0.012	*Nakamurella*	0.00395	0.008	0.006	0.020	0.015
0.012	Uncultured *Rhizobiaceae*	0.00298	0.012	0.041	0.000	0.000
0.011	*Flavobacterium*	0.19093	0.011	0.017	0.011	0.010
0.010	*Lactobacillus*	0.00298	0.011	0.013	0.001	0.001

*Note*: Statistical significance was tested using the Kruskal–Wallis one‐way analysis of variance. The statistical significance cut‐off value (*p*) < 0.05 was used.

Abbreviations: OTU, operational taxonomic unit; SD, standard deviation.

### Temporal variation in microbial diversity among daycare providers

**FIGURE 5:**
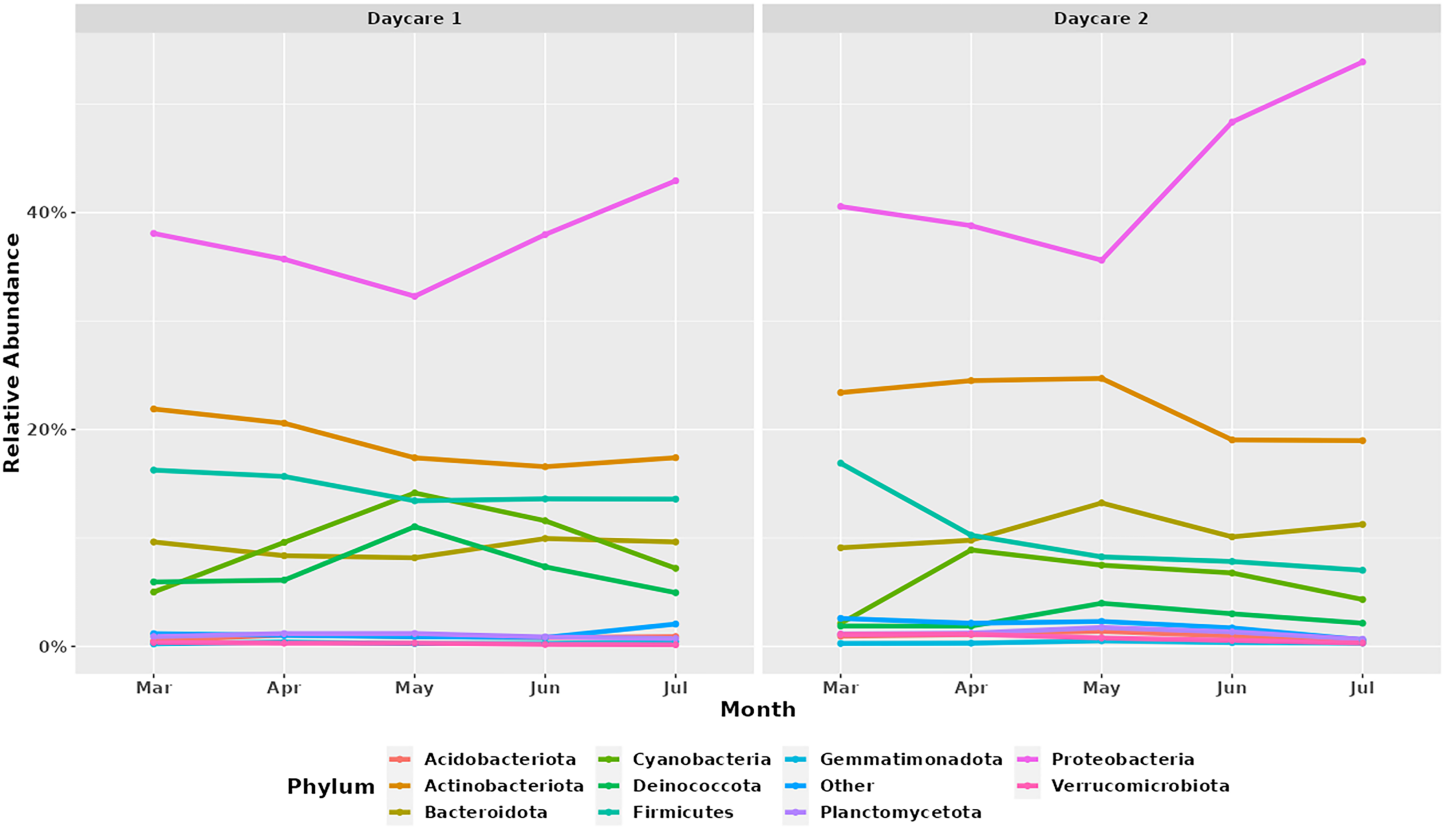
Temporal variation in the abundance of 10 most abundant phyla in (A) daycare 1 and (B) daycare 2.

Five consecutive samples were collected from two daycares between March and July 2021 and the abundance of different taxa was compared to detect any temporal differences. A minor decrease in the abundance of *Actinobacteriota*, *Bacteriodota*, *Firmicutes* and *Proteobacteria* between April and May was detected in samples from both daycare providers (Figure 5A,B). The abundance of *Proteobacteria* subsequently increased from June but not for the remaining three phyla. In contrast, the abundance of *Cyanobacteria* and *Deinococcota* slightly increased during April and May, followed by a reduction in June (Figure 5A,B). These results are consistent with the reports of seasonal changes in environmental microbiome in the daycare settings (Estensmo et al., 2021; Nunez et al., 2021; Prussin 2nd et al., 2016). A previous study found significant differences in air microbiome in urban settings between samples collected in Winter/Spring and those collected in Fall/Summer (Nunez et al., 2021). *Pseudomonas* species were more prevalent during the Spring, *Hymenobacter* during winter and *Nocardioides* and *Corynebacterium* during the Summer (Nunez et al., 2021). We also noticed minor changes in the abundance of these taxa between Spring and Summer samples along with other environment‐associated genera *Aliterella*, *Truepera* and uncultured *Chroococcidiopsaceae* (Table 2). However, corynebacteria were not in the top 20 genera showing differences in the abundance during these seasons. The abundance of *Acinetobacter* decreased in the samples from March and July from daycare 1, whereas it increased in samples from daycare 2 (Table 2). In particular, we did not observe any clear seasonal variations which is consistent with Prussin 2nd et al. (2016), who suggested that the level of occupancy has more influence on the microbial community structure in the daycare environment than the seasons (Prussin 2nd et al., 2016).

**TABLE 2:** Temporal variations in microbial abundances in dust samples from two daycare settings.

Taxon	Daycare 1					Daycare 2				
	Mar‐21	Apr‐21	May‐21	Jun‐21	Jul‐21	Mar‐21	Apr‐21	May‐21	Jun‐21	Jul‐21
*Acidiphilium*	0.012509	0.018566	0.016551	0.015496	0.024211	0.009406	0.009849	0.024615	0.012864	0.015835
*Acinetobacter*	0.043674	0.030235	0.024564	0.03181	0.023114	0.108555	0.089709	0.030983	0.16416	0.170949
*Aliterella*	0.024019	0.047948	0.068259	0.061579	0.029311	0.004419	0.006614	0.021191	0.020726	0.016011
*Bacillus*	0.009741	0.009382	0.006061	0.015477	0.008582	0.04018	0.017902	0.01161	0.009514	0.008551
*Blastococcus*	0.014974	0.018677	0.017567	0.01435	0.018085	0.00384	0.003839	0.00416	0.00342	0.0024
*Deinococcus*	0.013828	0.018096	0.018081	0.022748	0.013044	0.01019	0.008081	0.023667	0.015725	0.014349
*Flavobacterium*	0.015017	0.005464	0.002842	0.004748	0.005749	0.010258	0.008483	0.010339	0.0102	0.039202
*Hymenobacter*	0.016133	0.020717	0.01937	0.02608	0.024802	0.014972	0.012639	0.027392	0.018447	0.011201
*Massilia*	0.011901	0.006057	0.00436	0.011567	0.016373	0.008929	0.008699	0.02612	0.018365	0.010286
*Methylobacterium‐Methylorubrum*	0.015655	0.0178	0.009885	0.012202	0.01766	0.011189	0.012955	0.008097	0.007502	0.004343
*Nakamurella*	0.035586	0.016168	0.011905	0.012327	0.01753	0.013427	0.062073	0.019863	0.014562	0.007616
*Nocardioides*	0.032933	0.038344	0.036652	0.031078	0.020847	0.042815	0.040735	0.056601	0.038848	0.048293
*Paracoccus*	0.009045	0.00492	0.004201	0.005971	0.005867	0.004703	0.007017	0.003167	0.005839	0.001912
*Pseudomonas*	0.017974	0.012831	0.011049	0.009949	0.025888	0.029979	0.022345	0.011733	0.038813	0.046423
*Rubellimicrobium*	0.047994	0.06822	0.061764	0.062244	0.065032	0.023231	0.022071	0.043798	0.034195	0.026017
*Sphingomonas*	0.035876	0.042447	0.035876	0.043945	0.046274	0.034148	0.042475	0.046553	0.035114	0.026859
*Spirosoma*	0.019003	0.021323	0.025694	0.030838	0.026655	0.019584	0.023768	0.0444	0.028159	0.018806
*Streptococcus*	0.041717	0.040408	0.037394	0.032879	0.041057	0.016279	0.019656	0.010573	0.007816	0.005548
*Truepera*	0.045617	0.043041	0.092309	0.050658	0.0365	0.008611	0.010698	0.016138	0.014364	0.007045
Uncultured_*Chroococcidiopsaceae*	0.017467	0.03581	0.052472	0.028989	0.0278	0.004998	0.009921	0.02167	0.017435	0.015585
*Other*	0.519337	0.483548	0.443144	0.475066	0.505619	0.580286	0.56047	0.537329	0.483932	0.502769

It is important to note that the samples from daycare settings are limited in number (12 samples) in our study and we could not collect all seasons. Therefore, an analysis of larger sample size covering all seasons from wider cohort needs to be conducted to draw robust conclusions. However, our data are highly robust and reproducible. Two subsamples (D1 and D2) were collected from a single vacuum bag from one home to determine any bias introduced during the DNA extraction and technical replicates of all samples were included for sequencing to test the precision of sample sequencing. The subsamples and technical replicates clustered closely together in the Bray–Curtis dissimilarity NMDS ordination analysis (Figure S2), confirming the reproducibility and robustness of the dataset.

### Could exposure to microbial diversity in different environments help decrease the risk of childhood diseases?

The interaction with the environment early in childhood is key to improved health and immunity against NCDs (Chiu et al., 2017; Manichanh et al., 2006; Ott et al., 2004; Scher et al., 2015). Outdoor activities are linked with an increase in the microbial diversity on human skin and the respiratory tract (Selway et al., 2020). A biodiversity intervention led to an increase in the Gammaproteobacterial diversity in the environment and on the skin with enhanced immunoregulatory pathways in children (Roslund et al., 2020). In this study, we report various human‐ and environment‐associated taxa among the dust samples (Table 1, Figure 1), many with the potential to induce anti‐inflammatory and immune responses. For example, *Acinetobacter* species are known to induce anti‐inflammatory responses to environmental allergens (Fyhrquist et al., 2014). Skin commensal corynebacteria influence the skin immunity by promoting the activation of γδ T cells (Ridaura et al., 2018). Similarly, commensal staphylococci can raise skin immunity against colonization by opportunistic pathogen *Staphylococcus aureus* via antimicrobial production or signalling antagonism (Parlet et al., 2019). Commensal taxa may also induce immune response with cross reactivity against related pathogenic species, therefore, protecting from infections (Belkaid & Hand, 2014). For example, the oral commensal *Streptococcus mitis* induces antibodies that are cross‐reactive to pathogenic *Streptococcus pneumoniae* (Engen et al., 2014; Shekhar et al., 2019). Commensal lactobacilli have been reported to regulate adaptive and innate immunity by inducing T cells, natural killer cells and cytokines with anti‐inflammatory and immunomodulatory roles in the intestinal epithelium (Cristofori et al., 2021; Rastogi & Singh, 2022). We identified *Prevotella* species in dust samples, both from the home and daycare environments (Table 1). Some *Prevotella* species induce Th17 immune responses and production of various cytokines including IL‐10 which is an important predictive biomarker for acute lymphoblastic leukaemia (Larsen, 2017; Liu et al., 2020). *Prevotella* species were relatively less abundant in the gut microbiome of children with acute lymphoblastic leukaemia (Liu et al., 2020) and in the lung microbiome of patients with asthma and chronic obstructive pulmonary disease (Larsen, 2017). However, an elevated inflammatory response may also promote chronic inflammation among patients (Larsen, 2017). Several environmental taxa were more prevalent among the daycare samples (Table 1, Figures 1 and 3) and an exposure to common soil microbiota was associated with reduced risk of asthma (Winnicki et al., 2022). Therefore, the microbial diversity detected among the dust samples from both home and daycare environments may help decrease the risk of non‐communicable and infectious diseases among children.

## CONCLUSIONS

The microbial diversity among the dust samples collected from homes was relatively higher than in the daycare settings. Human‐associated taxa were more prevalent among the home samples, whereas environmental microbes which were previously reported to be associated with decreased risk of some NCDs are more abundant in dust samples from daycare settings. Therefore, an exposure to microbes in both settings is likely to help develop a better overall immunity to decrease the risk of childhood diseases than in a single setting. A lower diversity in the daycare samples and the observed variation in abundance of different taxa was potentially influenced by the COVID policies with more emphasis on personal hygiene, robust cleaning practices of shared spaces and limited indoor mixing. Several taxa detected in both environments are known to induce anti‐inflammatory and immunomodulatory responses and likely protect children from various non‐communicable and infectious diseases. The core taxa unique to the daycare setting may be responsible for the reported association between daycare attendance and the lower risk of some NCDs, but this needs further investigation. Further in‐depth temporal studies across different geographical regions are also warranted to understand differential environmental microbiomes. Such knowledge has the capacity to influence public health policy and guidance, as well as may lead to potential behavioural or probiotic/microbial prophylactic interventions to reduce the risks of NCDs such as childhood asthma and leukaemia.

## AUTHOR CONTRIBUTIONS

**Jill A. McKay:** Conceptualization (equal); funding acquisition (equal); methodology (equal); supervision (equal); writing – review and editing (equal). **Matthew Crown:** Formal analysis (equal); visualization (equal); writing – review and editing (equal). **Matthew Bashton:** Formal analysis (equal); methodology (equal); writing – review and editing (equal). **David Pearce:** Methodology (equal); writing – review and editing (equal). **Jane A. Entwistle:** Methodology (equal); writing – review and editing (equal). **Vartul Sangal:** Conceptualization (equal); data curation (equal); formal analysis (equal); funding acquisition (equal); methodology (equal); supervision (equal); writing – original draft (equal); writing – review and editing (equal).

## CONFLICT OF INTEREST STATEMENT

The authors declare no conflicts of interest.

## Supporting information


**Figure S1.** A rarefaction curve of all samples determines the minimum read depth for downstream analysis. Vertical line indicates the plateau at 40,952 reads.

**Figure S2.** Bray–Curtis dissimilarity NMDS ordination plot of technical replicates (sample IDs suffixed with A and B, respectively). D1 and D2 were subsamples collected from the same vacuum bag to detect any bias introduced during DNA extraction process. All replicates clustered closely together showing high reproducibility and robustness of the dataset.

**Figure S3.** Microbial composition of the dust samples from homes and daycares at the genus level. Twenty most prevalent genera are displayed, and the remaining genera are grouped as ‘other’.

**Table S1.** Details of dust samples collected from daycare providers and home settings.

## Data Availability

The sequencing data generated in this study are openly available from the European Nucleotide Archive via the study accession PRJEB46920 (individual sample accession numbers ERS17286601 to ERS17286681). URL: https://www.ebi.ac.uk/ena/browser/view/PRJEB70876
