# Supplementary figures and images for: Environmental microbiome in the home and daycare settings during the COVID‐19 pandemic, and potential risk of non‐communicable disease in children
Source: Environ Microbiol Rep. 2024 Jan 12;16(1):e13233. doi: 10.1111/1758-2229.13233 (PMC10866607; doi:10.1111/1758-2229.13233)

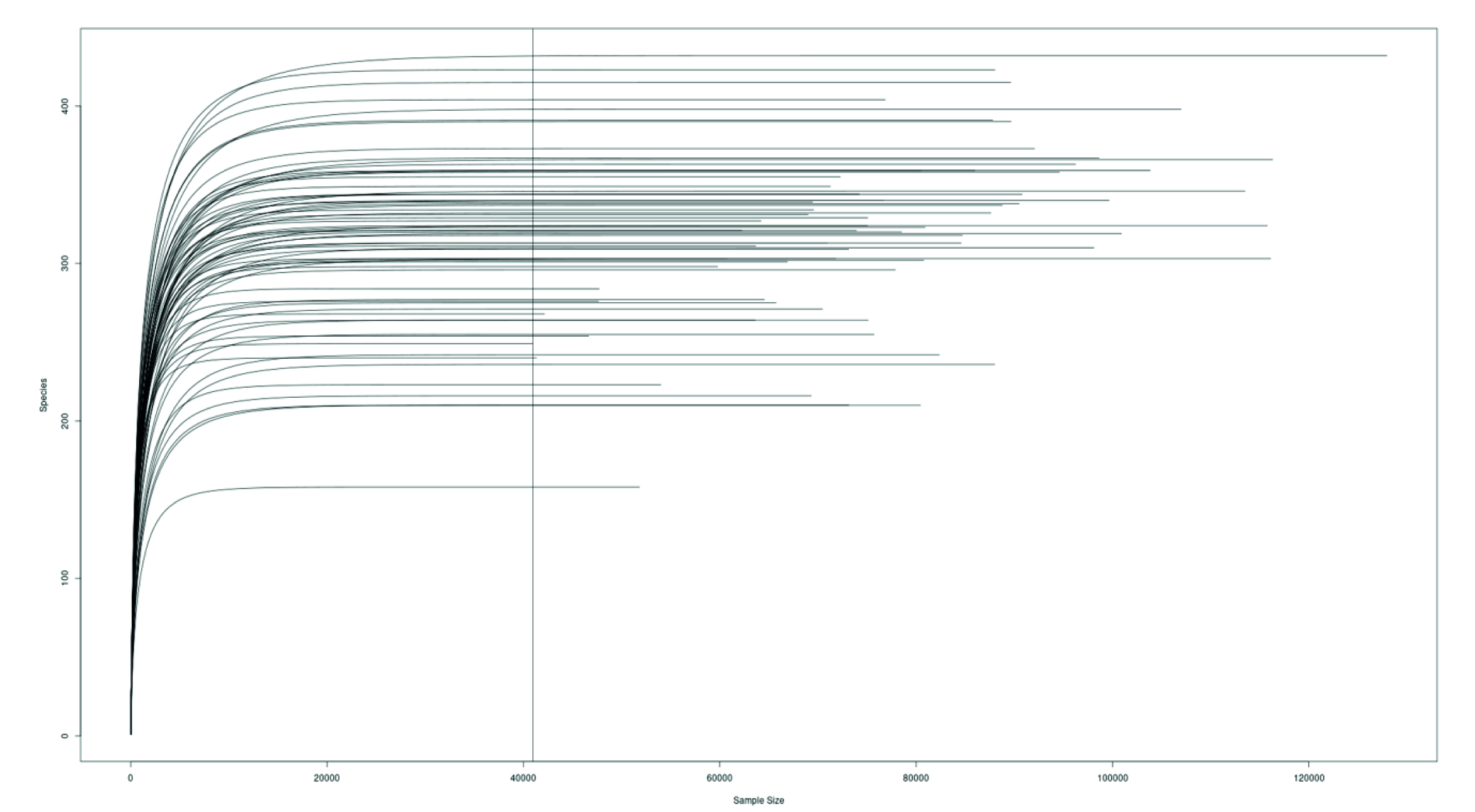

Supplement: Supplementary file 1 — Figure S1. A rarefaction curve of all samples determines the minimum read depth for downstream analysis. Vertical line indicates the plateau at 40,952 reads. [file EMI4-16-e13233-s002.tif]

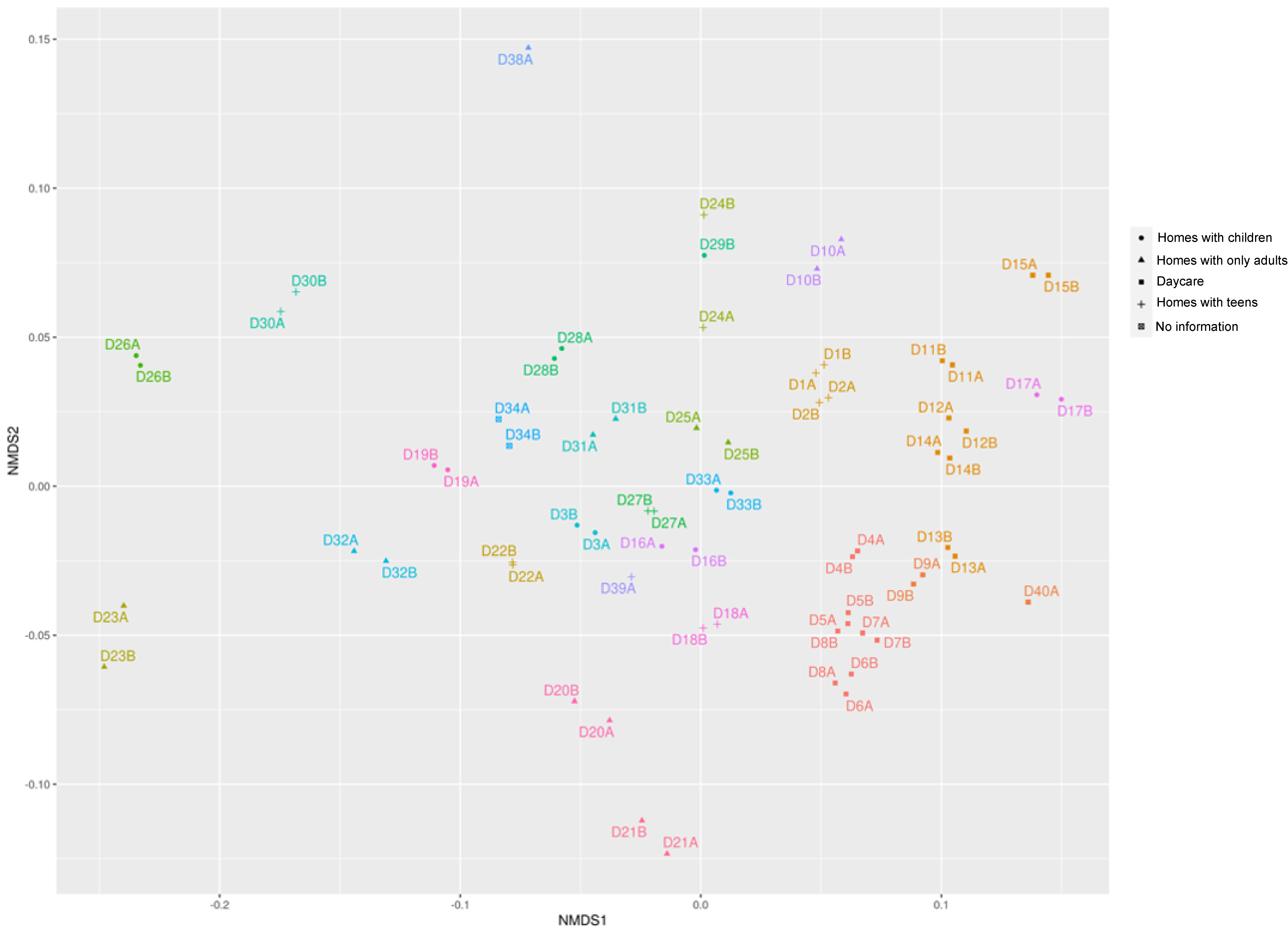

Supplement: Supplementary file 2 — Figure S2. Bray–Curtis dissimilarity NMDS ordination plot of technical replicates (sample IDs suffixed with A and B, respectively). D1 and D2 were subsamples collected from the same vacuum bag to detect any bias introduced during DNA extraction process. All replicates clustered closely together showing high reproducibility and robustness of the dataset. [file EMI4-16-e13233-s004.tif]

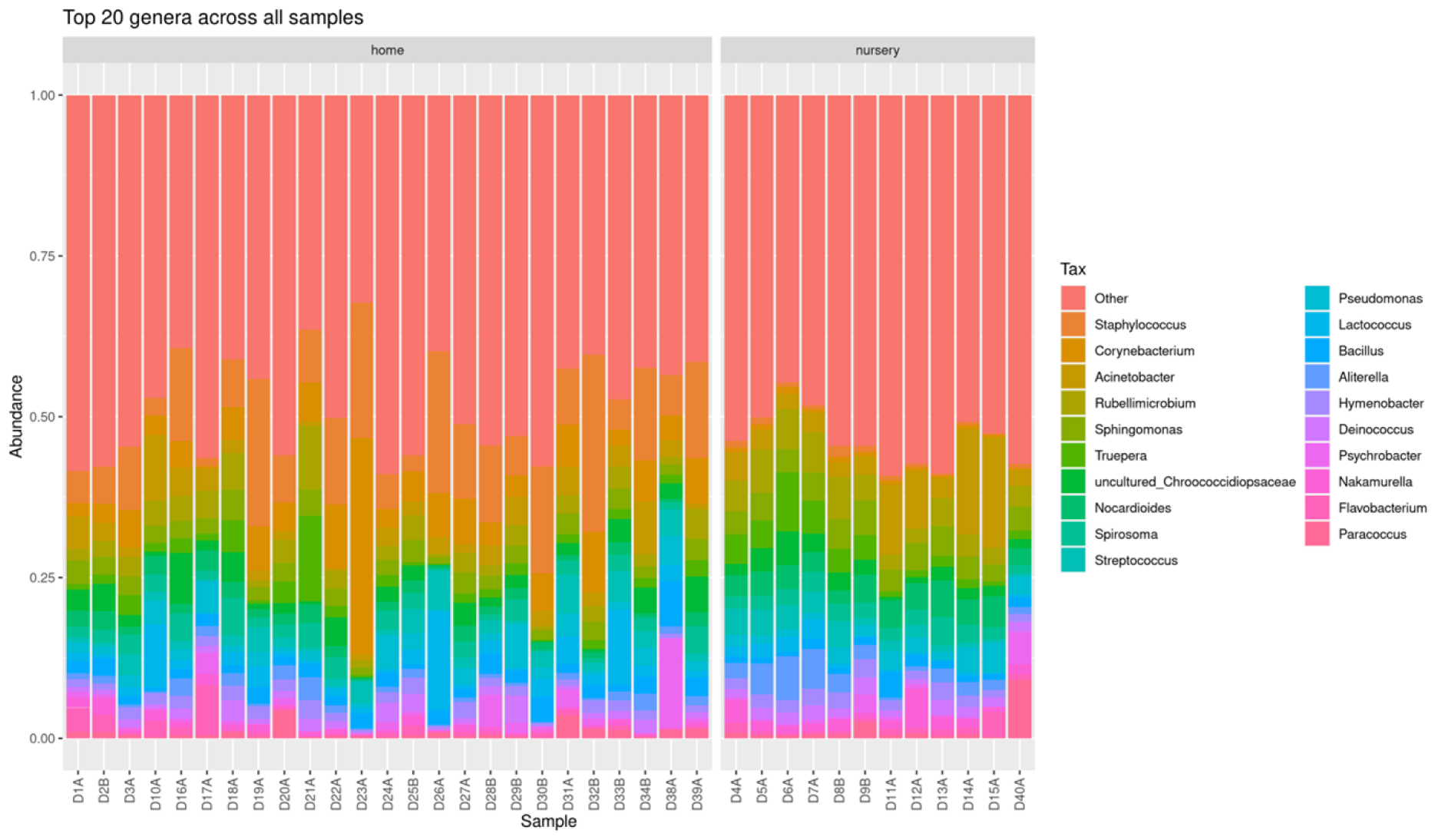

Supplement: Supplementary file 3 — Figure S3. Microbial composition of the dust samples from homes and daycares at the genus level. Twenty most prevalent genera are displayed, and the remaining genera are grouped as ‘other’. [file EMI4-16-e13233-s003.tif]
