# Supplementary material for: Environmental microbiome in the home and daycare settings during the COVID‐19 pandemic, and potential risk of non‐communicable disease in children
Source: Environ Microbiol Rep. 2024 Jan 12;16(1):e13233. doi: 10.1111/1758-2229.13233 (PMC10866607; doi:10.1111/1758-2229.13233)
Supplement: Supplementary file 4 — Table S1. Details of dust samples collected from daycare providers and home settings. [file EMI4-16-e13233-s001.docx]

Table S1. Details of dust samples collected from daycare providers and home settings.

|  |  |  |  | **Household information** | | | | | | |
| --- | --- | --- | --- | --- | --- | --- | --- | --- | --- | --- |
| **Sample** | **Type** | **Details** | **Collection Date** | **Total occupants** | **≤ 5 Y** | **6-10 Y** | **11-14 Y** | **15-17 Y** | **18- 65 Y** |  |
| D1 | H1_1 | Home | 04/04/2022 | 4 | 0 | 0 | 2 | 0 | 2 |  |
| D2 | H1_2 | Home | 04/04/2022 | 4 | 0 | 0 | 2 | 0 | 2 |  |
| D3 | H2 | Home | 04/04/2022 | 4 | 1 | 1 |  |  | 2 |  |
| D4 | DC1_1 | Daycare 1 | 24/03/2021 |  |  |  |  |  |  |  |
| D5 | DC1_2 | Daycare 1 | 27/04/2021 |  |  |  |  |  |  |  |
| D6 | DC1_3 | Daycare 1 | 11/05/2021 |  |  |  |  |  |  |  |
| D7 | DC1_4 | Daycare 1 | 02/06/2021 |  |  |  |  |  |  |  |
| D8 | DC1_5 | Daycare 1 | 21/07/2021 |  |  |  |  |  |  |  |
| D9 | DC2_1 | Daycare 3 | 22/06/2021 |  |  |  |  |  |  |  |
| D10 | H3 | Home | 29/03/2022 | 2 |  |  |  |  | 2 |  |
| D11 | DC3_1 | Daycare 2 | 23/03/2021 |  |  |  |  |  |  |  |
| D12 | DC3_2 | Daycare 2 | 23/04/2021 |  |  |  |  |  |  |  |
| D13 | DC3_3 | Daycare 2 | 27/05/2021 |  |  |  |  |  |  |  |
| D14 | DC3_4 | Daycare 2 | 25/06/2021 |  |  |  |  |  |  |  |
| D15 | DC3_5 | Daycare 2 | 19/07/2021 |  |  |  |  |  |  |  |
| D16 | H4 | Home | 01/05/2022 | 3 | 0 | 1 | 0 | 0 | 2 |  |
| D17 | H5 | Home | 13/05/2022 | 4 | 1 | 1 |  |  | 2 |  |
| D18 | H6 | Home | 06/06/2022 | 3 |  |  |  | 1 | 2 |  |
| D19 | H7 | Home | 20/05/2022 | 3 | 0 | 1 |  |  | 2 |  |
| D20 | H8 | Home | 23/05/2022 | 2 |  |  |  |  | 2 |  |
| D21 | H9 | Home | 23/05/2022 | 2 | 0 |  |  |  |  |  |
| D22 | H10 | Home | 16/05/2022 | 4 |  |  |  | 1 | 3 |  |
| D23 | H11 | Home | 13/05/2022 | 1 |  |  |  |  |  |  |
| D24 | H12 | Home | 16/06/2022 | 3 | 0 | 0 | 1 | 0 | 2 |  |
| D25 | H13 | Home | 11/05/2022 | 2 | 0 | 0 | 0 | 0 | 2 |  |
| D26 | H14 | Home | 11/05/2022 | 4 | 1 | 1 |  |  | 2 |  |
| D27 | H15 | Home | 04/05/2022 | 4 | 0 | 0 | 1 | 1 | 2 |  |
| D28 | H16 | Home | 24/05/2022 | 3 |  | 1 |  |  | 2 |  |
| D29 | H17 | Home | 18/05/2022 | 4 | 1 | 1 | 0 | 0 | 2 |  |
| D30 | H18 | Home | 06/06/2022 | 4 | 0 | 0 | 1 | 1 | 2 |  |
| D31 | H19 | Home | 07/06/2022 | 1 |  |  |  |  | 1 |  |
| D32 | H20 | Home | 21/06/2022 | 4 |  |  |  |  | 4 |  |
| D33 | H21 | Home | 21/06/2022 | 3 | 1 |  |  |  | 2 |  |
| D34 | H22 | Home | 21/06/2022 |  |  |  |  |  |  |  |
| D38 | H23 | Home | Jul-22 | 3 |  |  |  |  | 3 |  |
| D39 | H24 | Home | Jul-22 | 3 |  |  | 1 |  |  |  |
| D40 | DC2_2 | Daycare 3 | Jul-21 |  |  |  |  |  |  |  |
